# 3-(3-Nitro­benzyl­idene)pentane-2,4-dione

**DOI:** 10.1107/S1600536808043456

**Published:** 2008-12-24

**Authors:** Chuanbing Wu

**Affiliations:** a Department of Chemistry and Biology, Xiangfan University, Xiangfan 441053, People’s Republic of China

## Abstract

In the title mol­ecule, C_12_H_11_NO_4_, the two acetyl C—C=O planes are inclined to the benzene ring at angles of 18.03 (8) and 80.75 (7)°. In the crystal, adjacent mol­ecules are linked into centrosymmetric dimers by pairs of C—H⋯O inter­actions.

## Related literature

For metal-complexes with β-diketones, see: Youngme *et al.* (2007 ▶); Ma *et al.* (2005 ▶); Soldatov *et al.* (2003 ▶); Hinckley (1969 ▶).
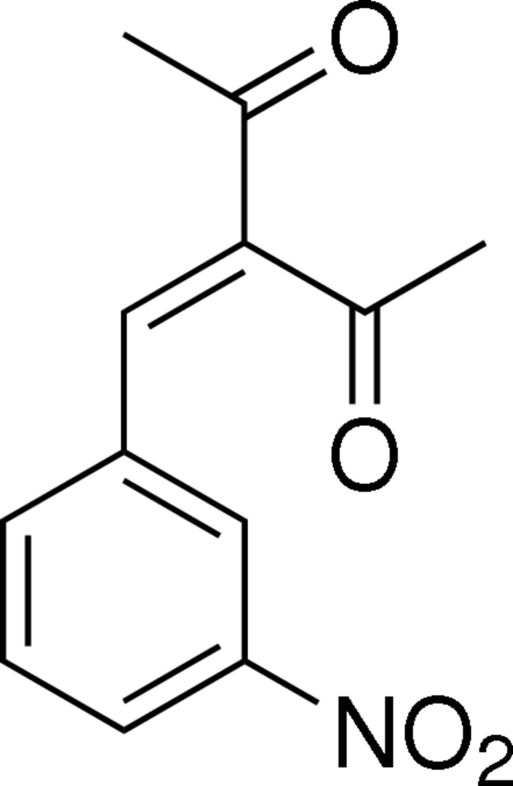


## Experimental

### 

#### Crystal data

C_12_H_11_NO_4_
*M* *_r_* = 233.22
Monoclinic, 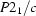

*a* = 16.0634 (9) Å
*b* = 5.2470 (3) Å
*c* = 14.9774 (9) Å
β = 114.117 (1)°
*V* = 1152.18 (12) Å^3^
*Z* = 4
Mo *K*α radiation
μ = 0.10 mm^−1^
*T* = 298 (2) K
0.20 × 0.10 × 0.10 mm

#### Data collection

Bruker SMART 4K CCD area-detector diffractometer
Absorption correction: none
4779 measured reflections
1998 independent reflections
1400 reflections with *I* > 2σ(*I*)
*R*_int_ = 0.048

#### Refinement

*R*[*F* ^2^ > 2σ(*F* ^2^)] = 0.068
*wR*(*F* ^2^) = 0.171
*S* = 1.07
1998 reflections
156 parameters
H-atom parameters constrained
Δρ_max_ = 0.21 e Å^−3^
Δρ_min_ = −0.17 e Å^−3^

### 

Data collection: *SMART* (Bruker, 1997 ▶); cell refinement: *SAINT* (Bruker, 1999 ▶); data reduction: *SAINT*; program(s) used to solve structure: *SHELXS97* (Sheldrick, 2008 ▶); program(s) used to refine structure: *SHELXL97* (Sheldrick, 2008 ▶); molecular graphics: *SHELXTL* (Sheldrick, 2008 ▶); software used to prepare material for publication: *SHELXTL*.

## Supplementary Material

Crystal structure: contains datablocks I, global. DOI: 10.1107/S1600536808043456/is2374sup1.cif

Structure factors: contains datablocks I. DOI: 10.1107/S1600536808043456/is2374Isup2.hkl

Additional supplementary materials:  crystallographic information; 3D view; checkCIF report

## Figures and Tables

**Table 1:** Hydrogen-bond geometry (Å, °)

*D*—H⋯*A*	*D*—H	H⋯*A*	*D*⋯*A*	*D*—H⋯*A*
C6—H6⋯O2^i^	0.93	2.42	3.282 (4)	153

Symmetry code: (i) 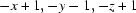.

**Fig. 1.**
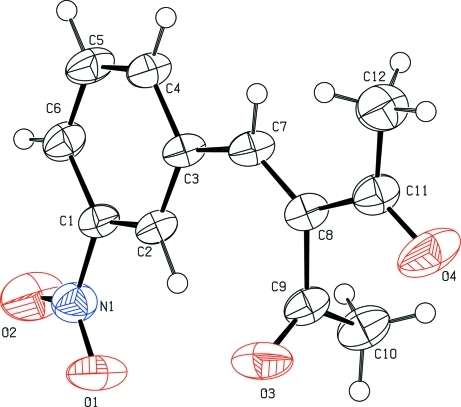
View of the title molecule, showing the atom-labelling scheme. The displacement ellipsoids are drawn at the 30% probability level.

## supplementary crystallographic information

### Comment

β-Diketone as an excellent chelating group has been widely used in supramolecular chemistry. It can form a variety of complexes with various transition-metals (*e.g.* Cu, Co, Ni, Mn, Pd, *etc*.) or rare-earth metals (*e.g.* Eu, Sm, La, Gd, *etc*.) (Youngme *et al.*, 2007; Ma *et al.*, 2005). These metal complexes have significant applications in material science or act as chemical shift reagents (Soldatov *et al.*, 2003; Hinckley, 1969). Herein, we prepared and crystallized 3-(3-nitrobenzylidene)pentane-2,4-dione, (I).

The title compound can be considered as an alkene having two CH_3_C(O)- substituents on one side of the double bond and the (NO_2_)C_6_H_3_– unit substituend on the other. The acetyl group *trans* to the H substituent is not coplanar with the double bond and the aromatic system as a twist is necessary to avoid crowding with the H atom of the aromatic ring.  The molecules are connected mainly by intermolecular C—H···O interactions.

### Experimental

Piperidine (0.85 g, 10 mmol) was added to a dimethylformamide solution (30 ml) of acetylacetone (1 ml, 10 mmol) and 3-nitrobenzaldehyde (1.51 g, 10 mmol). The mixture was heated at 413 K for 6 h. The mixture was poured into water (300 ml) and the organic phase was extracted with ethyl acetate. The ethyl acetate extract was dried over sodium sulfate and the solvent removed under reduced pressure to yield the crude product, which was recrystallized from ethanol to afford colourless crystals in 50% yield.

### Refinement

All H atoms were positioned geometrically (C—H = 0.93–0.96 Å) and refined as riding, allowing for free rotation of the methyl groups. The constraint *U*_iso_(H) = 1.2*U*_eq_(C) or 1.5*U*_eq_(C) (methyl C) was applied.

### Crystal data

**Table:**

C_12_H_11_NO_4_	*F*(000) = 488
*M**_r_* = 233.22	*D*_x_ = 1.344 Mg m^−^^3^
Monoclinic, *P*2_1_/*c*	Mo *K*α radiation, λ = 0.71073 Å
Hall symbol: -P 2ybc	Cell parameters from 1131 reflections
*a* = 16.0634 (9) Å	θ = 2.7–24.3°
*b* = 5.2470 (3) Å	µ = 0.10 mm^−^^1^
*c* = 14.9774 (9) Å	*T* = 298 K
β = 114.117 (1)°	Block, colorless
*V* = 1152.18 (12) Å^3^	0.20 × 0.10 × 0.10 mm
*Z* = 4	

### Data collection

**Table:**

Bruker SMART 4K CCD area-detector diffractometer	1400 reflections with *I* > 2σ(*I*)
Radiation source: fine-focus sealed tube	*R*_int_ = 0.048
graphite	θ_max_ = 25.0°, θ_min_ = 2.7°
φ and ω scans	*h* = −19→17
4779 measured reflections	*k* = −6→6
1998 independent reflections	*l* = −11→17

### Refinement

**Table:**

Refinement on *F*^2^	Primary atom site location: structure-invariant direct methods
Least-squares matrix: full	Secondary atom site location: difference Fourier map
*R*[*F*^2^ > 2σ(*F*^2^)] = 0.068	Hydrogen site location: inferred from neighbouring sites
*wR*(*F*^2^) = 0.171	H-atom parameters constrained
*S* = 1.07	*w* = 1/[σ^2^(*F*_o_^2^) + (0.085*P*)^2^ + 0.2322*P*] where *P* = (*F*_o_^2^ + 2*F*_c_^2^)/3
1998 reflections	(Δ/σ)_max_ = 0.015
156 parameters	Δρ_max_ = 0.21 e Å^−^^3^
0 restraints	Δρ_min_ = −0.16 e Å^−^^3^

### Special details

**Table:**

Geometry. All e.s.d.'s (except the e.s.d. in the dihedral angle between two l.s. planes) are estimated using the full covariance matrix. The cell e.s.d.'s are taken into account individually in the estimation of e.s.d.'s in distances, angles and torsion angles; correlations between e.s.d.'s in cell parameters are only used when they are defined by crystal symmetry. An approximate (isotropic) treatment of cell e.s.d.'s is used for estimating e.s.d.'s involving l.s. planes.
Refinement. Refinement of *F*^2^ against ALL reflections. The weighted *R*-factor *wR* and goodness of fit *S* are based on *F*^2^, conventional *R*-factors *R* are based on *F*, with *F* set to zero for negative *F*^2^. The threshold expression of *F*^2^ > σ(*F*^2^) is used only for calculating *R*-factors(gt) *etc*. and is not relevant to the choice of reflections for refinement. *R*-factors based on *F*^2^ are statistically about twice as large as those based on *F*, and *R*- factors based on ALL data will be even larger.

### Fractional atomic coordinates and isotropic or equivalent isotropic displacement parameters (Å^2^)

**Table:**

	*x*	*y*	*z*	*U*_iso_*/*U*_eq_	
C1	0.37414 (19)	−0.0732 (5)	0.46603 (19)	0.0328 (7)	
C2	0.31796 (19)	0.1238 (5)	0.4642 (2)	0.0359 (7)	
C3	0.30722 (19)	0.1886 (5)	0.54900 (19)	0.0348 (7)	
C4	0.3576 (2)	0.0531 (6)	0.6342 (2)	0.0405 (8)	
C5	0.4149 (2)	−0.1425 (6)	0.6345 (2)	0.0458 (8)	
C6	0.4239 (2)	−0.2098 (6)	0.5501 (2)	0.0399 (8)	
C7	0.2427 (2)	0.3821 (5)	0.5535 (2)	0.0389 (8)	
C8	0.1758 (2)	0.5058 (5)	0.4812 (2)	0.0361 (7)	
C9	0.1551 (2)	0.4824 (5)	0.3737 (2)	0.0374 (7)	
C10	0.0895 (2)	0.2825 (6)	0.3165 (2)	0.0536 (9)	
C11	0.1120 (2)	0.6808 (6)	0.5006 (2)	0.0434 (8)	
C12	0.1252 (3)	0.7457 (7)	0.6020 (2)	0.0606 (10)	
H2	0.2871	0.2142	0.4066	0.043*	
H4	0.3524	0.0957	0.6919	0.049*	
H5	0.4478	−0.2299	0.6923	0.055*	
H6	0.4621	−0.3426	0.5496	0.048*	
H7	0.2494	0.4254	0.6163	0.047*	
H10A	0.0808	0.2920	0.2492	0.080*	
H10B	0.0322	0.3087	0.3211	0.080*	
H10C	0.1132	0.1177	0.3423	0.080*	
H12A	0.0831	0.8774	0.6004	0.091*	
H12B	0.1865	0.8045	0.6380	0.091*	
H12C	0.1146	0.5971	0.6333	0.091*	
O1	0.34549 (17)	−0.0057 (5)	0.30328 (15)	0.0605 (7)	
O2	0.4208 (2)	−0.3406 (5)	0.37212 (17)	0.0735 (9)	
O3	0.19108 (17)	0.6251 (4)	0.33704 (16)	0.0612 (7)	
O4	0.04744 (19)	0.7640 (5)	0.43075 (17)	0.0764 (9)	
N1	0.38064 (17)	−0.1449 (5)	0.37404 (18)	0.0446 (7)	

### Atomic displacement parameters (Å^2^)

**Table:**

	*U*^11^	*U*^22^	*U*^33^	*U*^12^	*U*^13^	*U*^23^
C1	0.0375 (16)	0.0307 (15)	0.0255 (15)	−0.0020 (13)	0.0081 (12)	0.0000 (12)
C2	0.0389 (18)	0.0356 (17)	0.0271 (15)	0.0044 (14)	0.0073 (13)	0.0059 (13)
C3	0.0415 (18)	0.0329 (16)	0.0252 (15)	0.0022 (14)	0.0086 (13)	0.0008 (12)
C4	0.0454 (19)	0.0436 (18)	0.0279 (16)	0.0054 (16)	0.0101 (14)	0.0034 (13)
C5	0.053 (2)	0.0437 (19)	0.0331 (18)	0.0134 (16)	0.0098 (15)	0.0120 (14)
C6	0.0411 (18)	0.0362 (17)	0.0372 (17)	0.0087 (14)	0.0106 (14)	0.0055 (13)
C7	0.0489 (19)	0.0360 (17)	0.0276 (15)	0.0043 (15)	0.0113 (14)	0.0002 (13)
C8	0.0436 (18)	0.0282 (15)	0.0318 (16)	0.0010 (14)	0.0106 (13)	−0.0005 (12)
C9	0.0459 (19)	0.0255 (15)	0.0337 (17)	0.0109 (14)	0.0090 (14)	0.0030 (13)
C10	0.060 (2)	0.049 (2)	0.0380 (18)	−0.0040 (17)	0.0067 (16)	−0.0081 (15)
C11	0.051 (2)	0.0351 (17)	0.0397 (18)	0.0093 (16)	0.0144 (16)	0.0029 (15)
C12	0.067 (2)	0.069 (2)	0.049 (2)	0.024 (2)	0.0270 (19)	−0.0043 (18)
O1	0.0875 (19)	0.0624 (16)	0.0339 (13)	0.0208 (14)	0.0272 (13)	0.0112 (12)
O2	0.099 (2)	0.0719 (18)	0.0522 (15)	0.0440 (16)	0.0329 (14)	−0.0027 (13)
O3	0.0875 (19)	0.0539 (15)	0.0387 (13)	−0.0124 (14)	0.0223 (13)	0.0093 (11)
O4	0.085 (2)	0.080 (2)	0.0463 (15)	0.0496 (16)	0.0092 (14)	0.0050 (13)
N1	0.0504 (17)	0.0471 (16)	0.0372 (15)	0.0072 (14)	0.0187 (13)	−0.0009 (13)

### Geometric parameters (Å, °)

**Table:**

C1—C6	1.384 (4)	C8—C9	1.511 (4)
C2—C1	1.365 (4)	C9—C10	1.485 (4)
C2—H2	0.9300	C10—H10A	0.9600
C3—C2	1.392 (4)	C10—H10B	0.9600
C3—C4	1.394 (4)	C10—H10C	0.9600
C3—C7	1.472 (4)	C11—C12	1.486 (4)
C4—C5	1.377 (4)	C12—H12A	0.9600
C4—H4	0.9300	C12—H12B	0.9600
C5—C6	1.376 (4)	C12—H12C	0.9600
C5—H5	0.9300	O3—C9	1.206 (4)
C6—H6	0.9300	O4—C11	1.214 (3)
C7—H7	0.9300	N1—O1	1.219 (3)
C8—C7	1.341 (4)	N1—O2	1.219 (3)
C8—C11	1.490 (4)	N1—C1	1.472 (4)
			
C1—C6—H6	121.2	C9—C10—H10A	109.5
C1—C2—C3	119.6 (3)	C9—C10—H10B	109.5
C1—C2—H2	120.2	C9—C10—H10C	109.5
C2—C3—C4	117.9 (3)	C10—C9—C8	117.8 (3)
C2—C3—C7	124.2 (2)	C11—C8—C9	112.9 (2)
C2—C1—C6	122.8 (3)	C11—C12—H12A	109.5
C2—C1—N1	118.3 (2)	C11—C12—H12B	109.5
C3—C2—H2	120.2	C11—C12—H12C	109.5
C3—C7—H7	114.9	C12—C11—C8	121.2 (3)
C3—C4—H4	119.3	H10A—C10—H10B	109.5
C4—C3—C7	117.8 (3)	H10A—C10—H10C	109.5
C4—C5—H5	119.7	H10B—C10—H10C	109.5
C5—C6—C1	117.7 (3)	H12A—C12—H12B	109.5
C5—C6—H6	121.1	H12A—C12—H12C	109.5
C5—C4—C3	121.4 (3)	H12B—C12—H12C	109.5
C5—C4—H4	119.3	O1—N1—O2	123.1 (3)
C6—C1—N1	118.9 (3)	O1—N1—C1	118.4 (2)
C6—C5—C4	120.6 (3)	O2—N1—C1	118.5 (3)
C6—C5—H5	119.7	O3—C9—C10	122.4 (3)
C7—C8—C11	121.9 (3)	O3—C9—C8	119.8 (3)
C7—C8—C9	125.2 (3)	O4—C11—C12	120.9 (3)
C8—C7—C3	130.1 (3)	O4—C11—C8	117.8 (3)
C8—C7—H7	114.9		
			
C2—C1—C6—C5	0.5 (5)	C7—C8—C11—O4	−172.3 (3)
C2—C3—C4—C5	−1.3 (5)	C7—C8—C11—C12	6.0 (5)
C2—C3—C7—C8	9.8 (5)	C9—C8—C11—O4	6.7 (4)
C3—C2—C1—C6	−1.9 (5)	C9—C8—C11—C12	−175.1 (3)
C3—C2—C1—N1	177.1 (2)	C9—C8—C7—C3	−4.2 (5)
C3—C4—C5—C6	−0.1 (5)	C11—C8—C7—C3	174.6 (3)
C4—C5—C6—C1	0.5 (5)	C11—C8—C9—O3	90.6 (4)
C4—C3—C2—C1	2.3 (4)	C11—C8—C9—C10	−88.8 (3)
C4—C3—C7—C8	−167.0 (3)	O1—N1—C1—C2	9.6 (4)
C7—C3—C4—C5	175.7 (3)	O1—N1—C1—C6	−171.4 (3)
C7—C8—C9—O3	−90.5 (4)	O2—N1—C1—C6	8.7 (4)
C7—C3—C2—C1	−174.5 (3)	O2—N1—C1—C2	−170.3 (3)
C7—C8—C9—C10	90.1 (4)	N1—C1—C6—C5	−178.5 (3)

### Hydrogen-bond geometry (Å, °)

**Table:**

*D*—H···*A*	*D*—H	H···*A*	*D*···*A*	*D*—H···*A*
C6—H6···O2^i^	0.93	2.42	3.282 (4)	153

Symmetry codes: (i) −*x*+1, −*y*−1, −*z*+1.
